# Validation of the Chinese version of the Care Evaluation Scale for measuring the quality of structure and process of end-of-life care from the perspective of bereaved family

**DOI:** 10.1186/s12904-021-00777-4

**Published:** 2021-06-22

**Authors:** Juanjuan Zhao, Liming You, Hongmei Tao, Frances Kam Yuet Wong

**Affiliations:** 1 School of Nursing, Sun Yat-sen University, Guangzhou, China; 2 Department of Nursing, The Fifth Affiliated Hospital of Sun Yat-sen University, Zhuhai, China; 3 Faculty of Health & Social Sciences, School of Nursing, The Hong Kong Polytechnic University, Hunghom, Hong Kong SAR China

**Keywords:** Care Evaluation Scale, Validation, End-of-life care, Cancer, Structure and process, Bereaved family

## Abstract

**Background:**

Assessing the quality of structure and process of end-of-life care can help improve outcomes. There was currently no valid tool for this purpose in Mainland China. The aim of this study is to validate the Chinese version of the Care Evaluation Scale (CES).

**Methods:**

From January to December 2017, a cross-sectional online survey was conducted among bereaved family members of cancer patients from 10 medical institutes. The reliability of the CES was assessed with Cronbach’s α, and structural validity was evaluated by confirmatory factor analysis. Concurrent validity was tested by examining the correlation between the CES total score and overall satisfaction with end-of-life care, quality of dying and death, and quality of life.

**Results:**

A total of 305 valid responses were analyzed. The average CES score was 70.7 ± 16.4, and the Cronbach’s α of the CES was 0.967 (range: 0.802–0.927 for the 10 domains). The fit indices for the 10-factor model of CES were good(root-mean-square error of approximation, 0.047; comparative fit index, 0.952; Tucker–Lewis index, 0.946; standardized root mean square residual, 0.053). The CES total score was highly correlated with overall satisfaction with medical care (*r* = 0.775, *P* < 0.01), and moderately correlated with patients’ quality of life (*r* = 0.579, *P* < 0.01) and quality of dying and death (*r* = 0.570, *P* < 0.01). In addition, few associations between CES total score and demographic characteristics, except for the family members’ age.

**Conclusions:**

The Chinese version of the CES is a reliable and valid tool to evaluate the quality of structure and process of end-of-life care for patients with cancer from the perspective of bereaved family in Mainland China.

**Supplementary Information:**

The online version contains supplementary material available at 10.1186/s12904-021-00777-4.

## Background

Over the past decades, increasing attention has been given to the quality of end-of-life care with the rise of the hospice movement and the development of palliative care. According to an World Health Organization report published in 2014, palliative care is “an approach that improves the quality of life of patients and their families facing the problem associated with life-threatening illness, through the prevention and relief of suffering by means of early identification and impeccable assessment and treatment of pain and other problems, physical, psychosocial and spiritual.” [1] As mentioned in this definition, the goal of end-of-life care is more focused on quality of life. For patients facing death, the quality of dying and death is more important than the length of life [2]. Thus, understanding the experience of end-of-life care from both the perspective of patients and their families can help medical institutions identify and address the unmet needs of dying patients and improve care quality.

Reliable, valid, and clinically manageable measurement tools for evaluating the quality of care provided to dying patients are essential for improvements to be made [3]. Such tools should cover the experiences of patients and their families, as both should be the focus of high-quality end-of-life care [4]. Although feedback from dying patients is important, they may be unwilling or unable to answer questions about their care because of the deterioration of their physical and/or mental conditions [3]. Therefore, family members are an important source of information and several studies have proven that it is feasible and valid to evaluate the outcomes of end-of-life care from the perspective of bereaved families [5–7].

The quality of medical care can be measured in terms of structure (the environment in which healthcare is provided), process (the method by which healthcare is provided), and outcome (the consequences of healthcare) [8]. Several tools have been developed for measuring the quality of end-of-life care, but most of these evaluate outcomes [9–12] and few are designed to assess structure and process [10]. Some tools include evaluations of the structure/process of care, but their focus is limited to shared decision-making [12] and interpersonal skills and availability of physicians and nurses [11] while other important aspects (e.g. environmental and economic factors, psychosocial care for family members, etc.) are not covered [11, 13]. Although outcomes are important, the quality of structure and process is the basis for ensuring good care outcomes. More importantly, it is less influenced by patient- or family-related factors that healthcare providers cannot change [14–16].

The Care Evaluation Scale (CES), developed by Japanese researchers, is a tool to assess the quality of the structure and process of end-of-life care for patients with cancer [16]. After improving the language of the response options on the questionnaire, a revised version of CES 2.0 was established with good reliability and validity [7]. Cancer is the leading cause of disease-related death in both urban and rural areas in Mainland China [17], which means cancer patients are the largest disease group requiring end of life care in our country. The quality of medical care for patients with advanced cancer urgently need to be improved. A validated tool is key to help improve the quality of care through measuring and identifying the unmet needs of the patients and their families. With the purpose, in the present study we translated the English version of CES 2.0 into Chinese and evaluated its psychometric characteristics from the perspective of the bereaved family of cancer patients.

## Methods

### Measurements

The CES consists of 28 items in the following 10 domains: physical care by physicians, physical care by nurses, psycho-existential care, physician’s explanations to the patient, physician’s explanations to the family, environment, cost, consideration of family health, availability, and coordination and consistency [7]. Each item is scored using a 6-point Likert scale (1 = highly disagree, 2 = disagree, 3 = somewhat disagree, 4 = somewhat agree, 5 = agree, and 6 = highly agree). Participants were asked to select “7: N/A” if none of the other scores were applicable [7]. Domain scores were calculated as an average of the items in each domain, and the total score was calculated as an average of all domain scores. Scores were proportionally adjusted to range from 0 to 100 to facilitate interpretation, with a higher score indicating higher quality of care [7, 16].

### Translation and crosscultural adaptation

The crosscultural adaptation of the CES was conducted according to the guidelines of the American Association of Orthopaedic Surgeons Outcomes Committee [18]. With permission from the original authors, the forward and backward translations of the English version of CES were completed by two doctoral students and two postdoctoral research fellows respectively. A comprehensive version of the scale was submitted to an expert committee that included a professor of nursing education, professor of health management, specialist oncology nurse, clinical nurse manager, senior lecturer in medical English, and professor of rehabilitation medicine to evaluate the semantic equivalence and relevance of the content. The average scale-level content validity index and item-level content validity index were both 1.0. The proportion of semantic equivalence was 94.0 % (range: 83.3–100.0 %). The expert committee suggested no major change nor corrections on the item content and response options, but suggested minor amendments to statements of some items in the use of words and expressions that were comprehensible to the Mainland Chinese. A final Chinese version of CES was then formed. A pilot study was carried out among 49 bereaved family members of patients with cancer. Feedback from the participants confirmed that the scale items were easy to understand and can be completed in about ten minutes.

### Participants

We conducted a cross-sectional online survey from January to December 2017 to validate the Chinese versions of the CES and Good Death Inventory [19]. Participants were recruited by convenience sampling from 10 medical institutes including 7 oncology departments in general hospitals, 1 cancer center, 1 community hospital, and 1 palliative care unit of a general hospital, all of which provide end-of-life cancer care. The minimum sample size was estimated as 140 based on a minimum subject-to-item ratio of 5:1 to meet the requirements of factor analysis [20].

The inclusion criteria were as follows: deceased cancer patients aged ≥ 18 years who had been hospitalized for ≥ 72 h; bereaved family members aged ≥ 18 years who were the self-identified main caregivers of the patients, had basic ability in Chinese reading and writing and were able to complete the questionnaire [19]. The exclusion criteria were: patients died in the intensive care unit or died of treatment-related conditions (e.g. surgical complication, severe drug allergy, etc.); bereaved family members who could not be contacted by telephone or refused to participate in the study [19].

### Procedures and questionnaires

We recruited and trained one research assistant nurse at each institution. They made call to the potential bereaved family members, explained the survey purpose and recorded a valid phone number or email address for those who agreed to participate. A text message or email that included a website link of the survey materials was then sent to the participants by the principal investigator. The research assistant nurse would gave a reminding call to those participants who did not returning survey questionnaires. In addition to consent form and demographic information, the questionnaires also included the Chinese version of the CES and three general questions: “Were you satisfied overall with the medical care that the patient received during his/her last days?” (0 = absolutely dissatisfied to 10 = absolutely satisfied); “Based on your experience, how would you rate the overall quality of dying and death of the patient in the final moments?”; and “Based on your experience, how would you rate the overall quality of life of the patient in his/her last month of life?” (0 = terrible experience to 10 = almost perfect) [19].

### Ethics approval and consent to participate

 The study was approved by the Ethical Committee of School of Nursing, Sun Yat-sen University ,Guangzhou, China(2017ZSLYEC-004) and the ethics committees of all participating medical institutions. All participants had signed the informed consent documents.

### Statistical analysis

Data were analyzed using Mplus 7.0 [21] and SPSS 20.0. A significance level of 0.05 (2-sided test) was applied. The response “7: N/A” was treated as a missing value, which was replaced by the mean score of the item during the statistical process. Descriptive statistics were used to illustrate the demographic characteristics of patients and family members, clinical characteristics of patients, and CES scores. Analysis of variance or the Student’s t test was used to analyze variations in CES total score according to the characteristics of patients and bereaved family members. A Cronbach’s α value ≥ 0.70 was considered to indicate satisfactory internal consistency of the CES [22]. Confirmatory factor analysis (CFA) was used to evaluate the fit of the 10-factor CES model to the data. Four indices were adopted to evaluate the goodness-of-fit of the model: the root mean square error of approximation (RMSEA), standardized root mean square residual (SRMR), comparative fit index (CFI) and Tucker–Lewis index (TLI). The criteria for a good fit were RMSEA and SRMR < 0.08, and CFI and TLI ≥ 0.90 [23]. Factor loading levels were classified as low (< 0.30), mid-range (0.30–0.59), or high (≥ 0.60) [24]. Pearson correlation analysis of CES total score and overall satisfaction with end-of-life care, overall quality of dying and death, and overall quality of life was performed for concurrent validation of the CES.

## Results

### Recruitment and response

We initially identified 1912 potential bereaved family members of cancer patients. Of these, 703 could not be contacted, 510 refused, and 699 agreed to participate in the study. We sent out 699 questionnaires, of which 313 were returned. Eight questionnaires were excluded because more than half of the data were missing. Thus, responses of 305 questionnaires were ultimately analyzed (effective response rate, 43.6 %). There were no missing values in all the questionnaire. Our study is an online survey. Respondents were reminded when the questionnaire was submitted if any item was left unfilled, and only when all items had been answered can the questionnaire be submitted successfully. As we previously reported [19], there were no significant differences in patients’ age and sex between the respondents, nonrespondents, and refusals.

### Characteristics of study participants

The mean age of the 305 patients was 65 ± 13 years, and most patients were male (62.3 %) (Table 1). Most of the patients died in the oncology unit of general hospitals (76.1 %). The average length of the last hospital stay was 35 days, and the average length after the patients’ death was 18 months. The mean age of bereaved family was 42.5 years, and most were female (55.7 %), highly educated (college or higher) (70.2 %), and had a per capita monthly family income of 5000 yuan (700 USD) (58.4 %). Family members included children, parents, or siblings (78.0 %) and spouses (19.3 %). They cared for the patients for an average of 7 days (range: 5–7 days) per week, and spent an average of 8 h (range: 3–12 h) daily with patients. Most of the patients (83 %) and family members (81 %) did not have religion.

**Table 1:** CES total score according to the characteristics of patients and bereaved family members (*N*=305)

Variable			CES total score	ANOVA/t-test
			Mean±SD	(***p*** value)
**Patients**				
Age, years (mean±SD, range)	64.5±12.6 (18–96)			0.661
18–44	20	(6.6)	70.7±15.7	
45–59	74	(24.3)	70.6±15.5	
60–74	142	(46.6)	69.7±17.0	
75–96	69	(22.6)	72.7±16.5	
Sex				0.897
Male	190	(62.3)	70.8±15.2	
Female	115	(37.7)	70.5±18.3	
Religion				0.573
Yes	52	(17.0)	71.8±18.0	
No	253	(83.0)	70.4±16.1	
Primary site of cancer				0.172
Head and neck	30	(9.8)	75.4±15.4	
Chest	116	(38.0)	70.4±19.2	
Abdomen	102	(33.4)	70.7±12.6	
Pelvis	30	(9.8)	71.5±18.5	
Blood	20	(6.6)	62.6±17.0	
Other	7	(2.3)	73.7±15.3	
Place of death				0.304
Oncology unit of general hospital	232	(76.1)	70.4±16.5	
Cancer center	20	(6.6)	66.2±18.2	
Community center	19	(6.2)	75.8±16.6	
Palliative care unit	34	(11.1)	72.1±14.7	
Hospital days (mean±SD, range)	35.0±42.7 (3–365)			
Months after death (mean±SD, range)	18.3±11.1 (1.6–42.9)			
**Bereaved family members**				
Age, years (mean±SD, range)	42.5±11.0 (19–84)			0.001
18–39①	121	(39.7)	67.2±15.6	①②*
40–49②	118	(38.7)	71.1±17.4	①③*
50–84③	66	(21.6)	76.4±14.5	
Sex				0.566
Male	135	(44.3)	70.1±17.7	
Female	170	(55.7)	71.2±15.4	
Education				0.154
High school or lower	91	(29.8)	72.7±18.4	
College	90	(29.5)	68.1±17.3	
University or higher	124	(40.7)	71.1±14.0	
Religion				0.840
Yes	58	(19.0)	71.1±19.5	
No	247	(81.0)	70.6±15.6	
Relationship to patient				0.139
Spouse	59	(19.3)	73.6±15.8	
Children/parent/sibling^a^	238	(78.0)	69.7±16.5	
Friend/colleague	8	(2.6)	77.4±16.7	
Per capita monthly family income (yuan)				0.620
<3000	45	(14.8)	72.7±15.3	
3000–4999	82	(26.9)	71.0±17.4	
≥5000	178	(58.4)	70.0±16.3	
Health status during caregiving period				0.960
Good	173	(56.7)	70.9±16.9	
Moderate	123	(40.3)	70.5±16.0	
Poor	9	(3.0)	69.6±13.8	
Average days of care per week, median (P25, P75)	7 (5, 7)			
Average hours spent with patient per day, median (P25, P75)	8 (3 ,12)			

Values are shown as n (%) unless otherwise indicated

*ANOVA* analysis of variance, *CES* Care Evaluation Scale, *P25* 25th percentile, *P75* 75th percentile, *SD* standard deviation

^a^Child (*n*=228), parent (*n*=4), and sibling (*n*=6)

①②③ group number of the family member’s age

^*^Significant differences detected using Tukey’s test

### CES scores

The average total score of the CES was 70.7 ± 16.4. (Table 2). Among the 10 domains, the highest score was for “physician’s explanations to the family” (77.4 ± 18.9), followed by “coordination and consistency” (76.2 ± 16.9) and “physical care by physician” (74.7 ± 20.7). The lowest scores were for “consideration of family health” (58.86 ± 25.58), “environment” (66.1 ± 22.2), “cost” (69.4 ± 20.1)”, and “physicians’ explanations to the patient” (69.4 ± 21.7).

**Table 2:** Reliability of the Care Evaluation Scale

Domain	Mean ± SD	Cronbach’s α^a^
I. Physical care by physician	74.7 ± 20.7	0.901
II. Physical care by nurse	74.3 ± 19.4	0.915
III. Psycho-existential care	70.2 ± 21.1	0.927
IV. Physician’s explanations to the patient	69.4 ± 21.7	0.898
V. Physician’s explanations to the family	77.4 ± 18.9	0.911
VI. Environment	66.1 ± 22.2	0.916
VII. Cost	69.4 ± 20.1	0.802
VIII. Consideration of family health	58.9 ± 25.6	0.915
IX. Availability	70.3 ± 19.7	0.826
X. Coordination and consistency	76.2 ± 16.9	0.856
Total score	70.7 ± 16.4	0.967

*SD* standard deviation

^a^Cronbach’s α > 0.70 indicate scale reliability

CES total score varied according to family members’ age (*P* = 0.001). Family members aged < 40 years gave lower CES scores for patients. There were no significant differences in CES total score according to patients’ age, sex, religion, diagnosis, or place of death or family members’ sex, education level, religion, income, or health status during the caregiving period (Table 1).

### Reliability

Internal consistency was evaluated with Cronbach’s α (Table 2). The Cronbach’s α for CES was 0.967, and ranged from 0.802 to 0.927 for the 10 domain scores.

### Construct validity

**Fig. 1:**
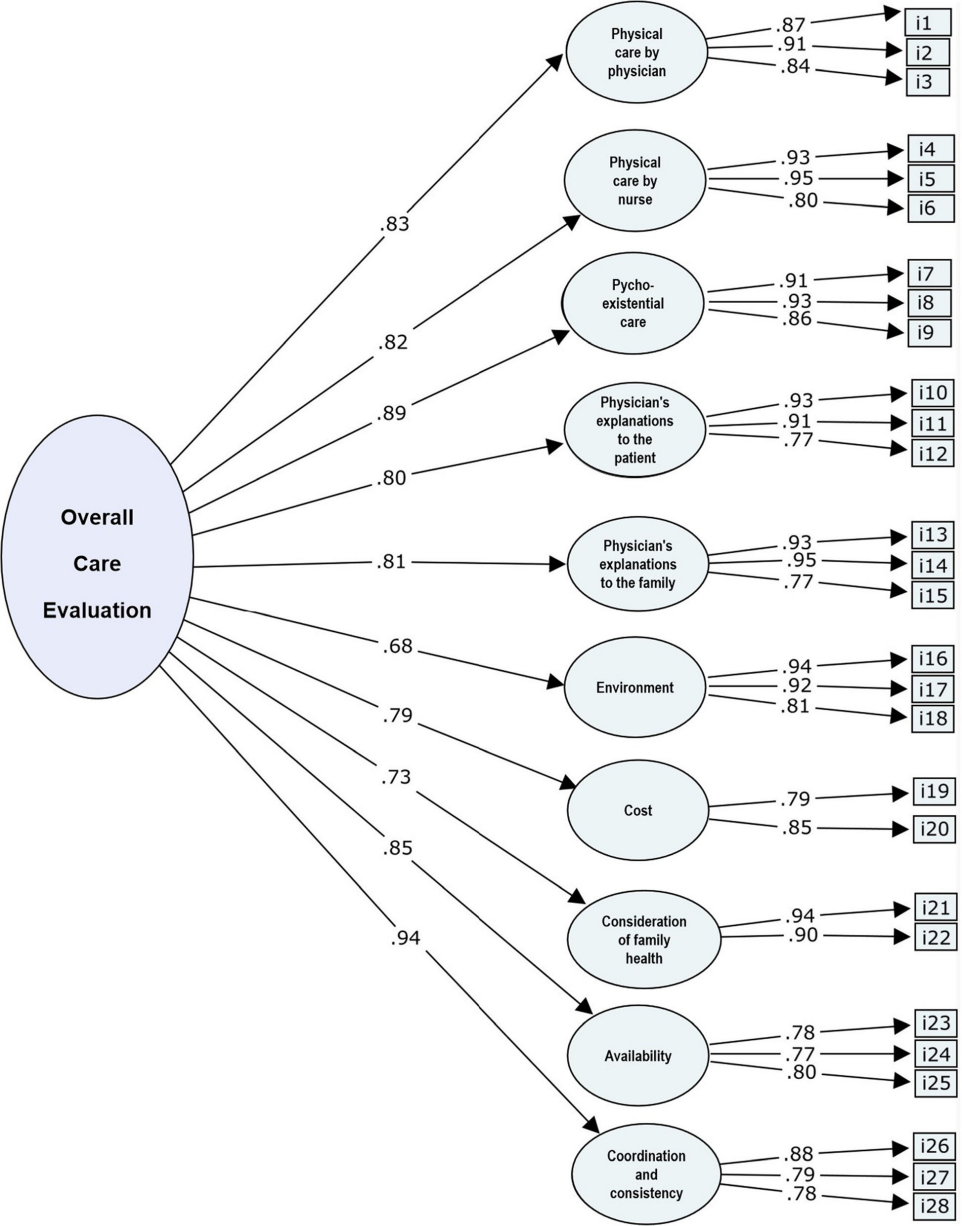
Structure of the Chinese version of the Care Evaluation Scale

The results of the CFA are shown in Fig. 1. All domains showed high factor loading (0.68–0.94), indicating a high correlation between the observed domains and overall care evaluation. The fit indices for the 10-factor model of CES were good (RMSEA = 0.047, CFI = 0.952, TLI = 0.946, and SRMR = 0.053).

### Concurrent validity

The results of the concurrent validity analysis are shown in Table 3. CES total score was highly correlated with overall satisfaction with medical care (*r* = 0.775, *P* < 0.01), and moderately correlated with patient’s quality of life (*r* = 0.579, *P* < 0.01) and quality of dying and death (*r* = 0.570, *P* < 0.01).

**Table 3:** Concurrent validity of the Care Evaluation Scale

Domains	Overall satisfaction	Overall quality of life	Overall quality of death and dying
I. Physical care by physician	0.649**	0.475**	0.490**
II. Physical care by nurse	0.702**	0.485**	0.473**
III. Psycho-existential care	0.676**	0.506**	0.484**
IV. Physician’s explanations to the patient	0.574**	0.434**	0.405**
V. Physician’s explanations to the family	0.602**	0.429**	0.414**
VI. Environment	0.571**	0.472**	0.464**
VII. Cost	0.529**	0.430**	0.437**
VIII. Consideration of family health	0.562**	0.436**	0.431**
IX. Availability	0.624**	0.471**	0.469**
X. Coordination and consistency	0.687**	0.473**	0.486**
**Total score**	0.775**	0.579**	0.570**

Values are Pearson’s correlation coefficients

**P < 0.01

## Discussion

In this study, we evaluated the psychometric properties of the Chinese version of the CES to measure quality of structure and process of end-of-life care from the prospective of bereaved family members of cancer patients in Mainland China. We found few associations between CES score and most demographic characteristics of patients and their family members, except for the latter’s age.

Our results demonstrated that the Chinese version of the CES had good internal reliability, structural validity, and concurrent validity. As for the reliability, the overall Cronbach’s α was 0.967 for the CES scale and ranged from 0.802 to 0.927 for the 10 domains, indicating good internal consistency between the items of the scale and those in each dimension. The original Japanese version of CES 2.0 [7], the Korean version [25] and the Spanish version [15] all showed good internal consistency (Japanese version: α = 0.96 for total CES score and α = 0.87–0.95 for the 10 domains; Korean version: α = 0.97 for total score and α = 0.88–0.94 for the domains; Spanish version: α = 0.90 for total score and α = 0.85–0.89 for the domains). Thus, the CES is stable when used in different Asian countries as well as the west culture.

To confirm the validity, first ,we conducted a CFA and found that the data in this study fit well with the 10-factor structure model of the original version of the CES 2.0 [7]. The standardized factor loading of each domain was > 0.60 (range: 0.68–0.94), indicating a strong correlation between each domain and overall quality of care and reflecting good structural validity. Then, we evaluated the concurrent validity of the CES by examining the correlation between CES total score and response to three general questions related to satisfaction regarding end-of-life care, quality of life, and quality of dying and death of the patients and found a significant positive correlation, which further confirmed the validity of the Chinese version of the CES.

In this study, the mean CES total score of patients with cancer in our study (70.7 ± 16.4) was comparable to that of Japanese patients with cancer who died without receiving palliative or hospice care (68 ± 21) [26], but lower than that of patients in Korea (73.77) [25] and Japan (78 ± 17 [26] and 80 ± 12 [16]) who had received such care until death. In a national study on quality of end-of-life cancer care conducted in Japan, bereaved family members reported that cancer patients who received neither palliative nor hospice care suffered greater physical symptom distress, highlighting an urgent need to improve both physicians’ and nurses’ physical care knowledge and skills [26]. Therefore, it is essential to establish and train palliative care teams at hospitals and increase the proportion of cancer patients treated by these teams.

In terms of the domain scores of the Chinese version of the CES, the highest score was for “physician’s explanations to the family” and the lowest was for “consideration of family health”. This result suggests that considerable support was provided to family members participating in medical decision-making for cancer patients, reflecting the universality and importance of their involvement in the end-of-life care of cancer patients in Mainland China [27, 28]. On the other hand, the physical and mental health of families of cancer patients was not given adequate attention, which is a key component of end-of-life cancer care and should be improved in the future.

Few demographic factors were associated with the rating of quality of end-of-life care structure and process, which is consistent with previous studies in which evaluations of care structure and process were not strongly influenced by patient- or family-related subjective factors such as the degree of care expectation, depression, or social desirability [16] or the patient’s general physical condition [14]. However, in our study, family members aged < 40 years gave lower scores for quality of care of cancer patients, which is in agreement with our previous finding that younger family members gave lower scores quality of death scores for these patients [19]. Given that quality of death is the outcome of end-of-life care, lower CES scores may reflect younger individuals’ difficulty in accepting death rather than a negative assessment of the quality of care.

To our knowledge, all current evidence on the development and application of the CES has been obtained from studies conducted on patients with cancer [7, 14–16, 25]. However, the prevalence of end-of-life care needs were similar in patients with non-cancer and cancer diagnoses [29, 30]. Also, the items of CES are not specific to cancer diagnosis but rather the quality of structure and process of end-of-life care. Therefore, we consider that the CES holds promise as a tool to assess the quality of end-of-life care in non-cancer patients.

This study had some limitations. Firstly, the test-retest reliability was not conducted, thus the stability of the Chinese version of the CES are still unknown and need to be further explored in the future. Secondly, most of the data were collected from the oncology departments of general hospitals. Thus, the results only reflect maximally the quality of structure and process of end-of-life cancer care in these hospitals and may not be generalizable to other types of institutions. Thirdly, although there were no differences in patients’ age and sex between respondents, nonrespondents and refusals, we have no access to detailed information on the latter 2 groups of the family members, which limits the representativeness of the sample. Fourthly, the average age of family members participating in this study was 42.5 years, over 70 % were highly educated, and nearly 60 % had a relatively high income; this demographic profile needs to be considered when the results of this study are generalized to populations with different characteristics. Lastly, the validation of CES was assessed among bereaved family of cancer patients, the applicability in non-cancer patients need to be further explored in the future.

## Conclusions

In conclusion, evaluation of the quality of care provided to dying patients are essential for making improvements to be made in the field of health service of end-of-life care. This study culturally adapted the Care Evaluation Scale which was originally developed in Japan. The results of our study demonstrated that the Chinese version of the CES is easy to understand and has good reliability and validity. In addition, we found few associations between CES score and most demographic characteristics of patients and their family members (except for the latter’s age), which gave evidence that the tool can objectively measure the quality of care without much influence of subjective characteristics of respondents. We conclude that the Chinese version of the CES is culturally appropriate and psychometrically reliable and valid to be used as a tool of evaluating quality of structure and process of end-of-life cancer care from the perspective of the bereaved family in Mainland China.

## Supplementary Information

**Additional file 1: Supplement 1.** The English version of the Care Evaluation Scale (CES)*

## Data Availability

The datasets during and/or analyzed during the current study and the Chinese version of CES are available from the corresponding author on reasonable request.

## Abbreviations

CES: Care Evaluation Scale
CFA: Confirmatory Factor Analysis
RMSEA: Root Mean Square Error of Approximation
SRMR: Standardized Root Mean Square Residual
CFI: Comparative Fit Index
TLI: Tucker–Lewis Index
ANOVA: Analysis of Variance
P25: 25th percentile
P75: 75th percentile
SD: Standard Deviation
