# Supplementary material for: Validation of the Chinese version of the Care Evaluation Scale for measuring the quality of structure and process of end-of-life care from the perspective of bereaved family
Source: BMC Palliat Care. 2021 Jun 22;20:85. doi: 10.1186/s12904-021-00777-4 (PMC8220706; doi:10.1186/s12904-021-00777-4)
Supplement: Supplementary file 1 — Additional file 1: Supplement 1. The English version of the Care Evaluation Scale (CES)* [file 12904_2021_777_MOESM1_ESM.docx]

**Supplement 1- The English version of the Care Evaluation Scale (CES)***

## Physical care by physician

## 1. Physicians endeavor to relieve patients’ physical discomfort.

## 2. Physicians deal promptly with patients’ discomforting physical symptoms.

## 3. Physicians have adequate knowledge and skills to alleviate patients’ physical symptoms.

## Physical care by nurse

## 4. Nurses endeavor to relieve patients’ physical discomfort.

## 5. Nurses deal promptly with patients’ discomforting physical symptoms.

## 6. Nurses have adequate knowledge and skills to alleviate patients’ physical symptoms.

## Psycho-existential care

## 7. Physicians, nurses, and staff endeavor to relieve patients’ concerns and worries.

## 8. Physicians, nurses, and staff endeavor to relieve patients’ sadness and depression.

## 9. Physicians, nurses, and staff endeavor to give hope to patients.

## Physicians’ explanations to patients

## 10. Physicians give sufficient explanations to patients regarding their condition and treatment.

## 11. Physicians give easy-to-understand explanations to patients regarding their condition and treatment.

## 12. Consideration is given so that patients will participate in the selection of treatment.

## Physicians’ explanations to families

## 13. Physicians give sufficient explanations to the family regarding the patient’s condition and treatment.

## 14. Physicians give easy-to-understand explanations to the family regarding the patient’s condition and treatment.

## 15. Consideration is given so that the family will participate in the selection of treatment.

## Environment

## 16. Hospital or room is convenient and comfortable.

## 17. Environment is quiet and calm.

## 18. Toilet and washstand facilities are convenient.

## Cost

## 19. The total cost was reasonable.

## 20. The content of the medical bills was easy to understand.

## Consideration of family health

## 21. Consideration was given to the health of the family.

## 22. Consideration was given so that the family could have their own time and continue to work.

## Availability

## 23. When necessary, admission (use) is possible without waiting.

## 24. The admission (use) procedures are simple.

## 25. Admission (use) is in accordance with the wishes of the patient and their family.

## Coordination and consistency

## 26. There is good cooperation among staff members such as physicians and nurses.

## 27. Important information is shared, even when the attending physician or nurse changes.

## 28. Treatment is planned with appropriate consideration of the previous course of the disease.

## *Note : The English version of CES was developed and permitted to be used in this study by Dr Mitsunori Miyashita. The Chinese version is available from the corresponding author of this study upon request.
